# Freshwater fish diversity of the Yongding River, the largest river flowing through Beijing

**DOI:** 10.3897/BDJ.13.e144995

**Published:** 2025-03-18

**Authors:** Chen Tian, Xuejian Li, Zhixian Sun, Chengyi Niu, Fushan Zheng, Yahui Zhao

**Affiliations:** 1 Key Laboratory of the Zoological Systematics and Evolution, Institute of Zoology, Chinese Academy of Sciences, Beijing, China Key Laboratory of the Zoological Systematics and Evolution, Institute of Zoology, Chinese Academy of Sciences Beijing China; 2 Institute of Shandong River Wetlands, Jinan, China Institute of Shandong River Wetlands Jinan China

**Keywords:** Beijing, ecological water replenishment, distribution, fish species

## Abstract

**Background:**

The Yongding River, the largest river flowing through Beijing, is a major tributary of the Haihe River system. It holds significant ecological, economic and social importance in northern China. However, due to the climate change and anthropogenic activities, parts of the tributaries and lower mainstream of the Yongding River have dried up in recent decades. To alleviate the water scarcity crisis of the Yongding River, ecological water replenishment was initiated in 2020 based on the Middle Route of the South-to-North Water Diversion Project and the Wanjiazhai Yellow River Diversion Project. As a result, the fish population structure and diversity of the Yongding River may change accordingly. In this context, before large-scale water replenishment started, we conducted two field surveys in the summer and autumn of 2019 to assess fish diversity in the Yongding River Basin.

**New information:**

This study provides information of 45 fish species belonging to nine orders, 18 families, seven subfamilies and 33 genera in the Yongding River Basin. Our study includes one dataset that presents taxonomy, distribution, water body and location for each species collected from the Yongding River. The provided data can assist other researchers in assessing the impact of water replenishment on aquatic biodiversity and the broader ecological environment of northern China.

## Introduction

The Yongding River, the largest tributary of the Haihe River system, is a crucial water source in northern China (Ding et al. 2022). This river system formed in the late Middle Pleistocene (Li 1988). After cutting through the Guanting Canyon, the Yongding River alluvialised to form most of the Beijing Plain landscape over the following tens of thousands years (Wang and Xu 1987, Li et al. 2023). The current length of the Yongding River is 759 km, with a drainage area of 47,000 km^2^ and an annual runoff of approximately 6.55 billion m^3^ (Lu et al. 2022). It originates from Shanxi Province and runs through the Inner Mongolia Autonomous Region, Hebei Province, Beijing and Tianjin Cities and eventually drains into the Haihe River (Niu et al. 2021, Lu et al. 2022). The upper reaches of the Yongding River are divided into two major tributaries: the Sanggan River in the south, which originates from Ningwu County, Shanxi Province; and the Yang River in the north, which originates from Xinghe County, Inner Mongolia Autonomous Region (Wang et al. 2020, Kong et al. 2021). This two tributaries join at Huailai County in Hebei Province, forming the mainstream of the Yongding River.

**Figure 1.**
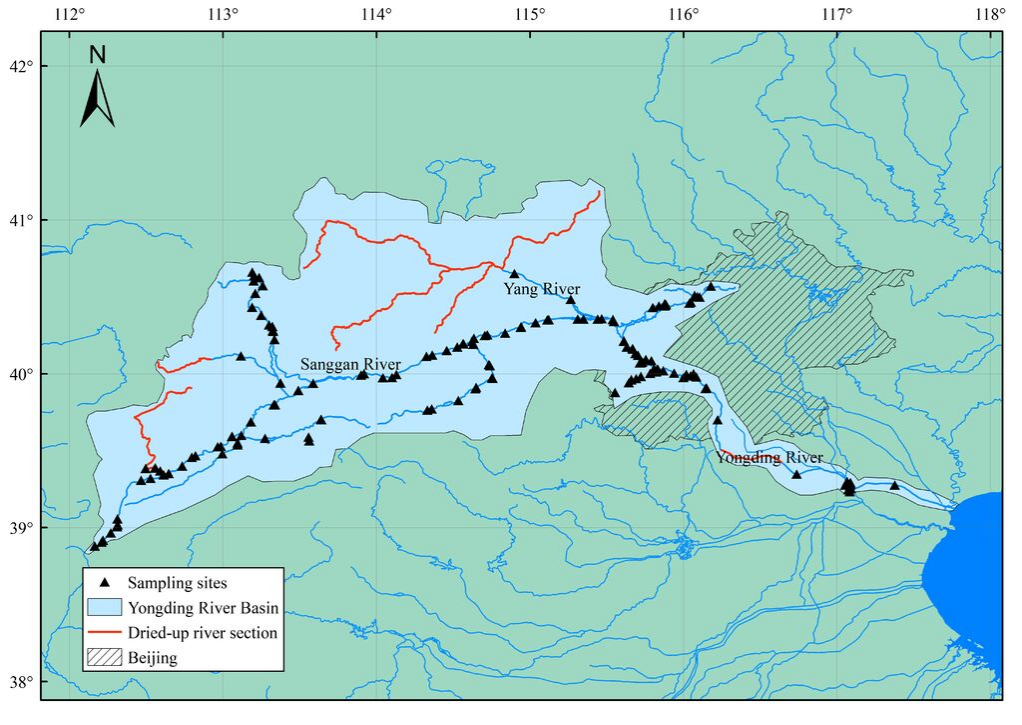
Location of the sampling sites.

Beijing, the capital of China, is a super metropolis settled on the Yongding River. This city covers an area of 16,410.54 km^2^, with a population exceeding 20 million (Wen et al. 2023, Li et al. 2023). The Yongding River Basin is one of the most important water conservation areas and water resources of Beijing and it is also the ecological barrier and corridor of Beijing and the adjacent areas (Men et al. 2015). However, in recent decades, as Beijing undergoes a significant urban expansion, water consumption has increased rapidly and the shortage of water resource is aggravated by decreasing precipitation in northern China (Peng et al. 2013, Zhou et al. 2014). Consequently, since 1996, sections of the Yongding River tributaries and the lower mainstream (Fig. 1) were in a state characterised by seasonal or long-term cutoff (Jiang et al. 2014).

In order to alleviate water scarcity crisis of Beijing and the adjacent areas, the Middle Route of the South-to-North Water Diversion Project officially started transferring water from the Danjiangkou Reservoir in the Yangtze River Basin to Beijing in 2014 (Zhu et al. 2021). The large-scale water replenishment of the Yongding River was initiated in 2020. In the same year, the Wanjiazhai Yellow River Diversion Project began recharging water to the Yongding River from the Wanjiazhai Reservoir in the Yellow River Basin (Li et al. 2020). By the end of 2023, the water replenishment to the Yongding River had surpassed 20 billion m^3^ (Bai et al. 2023, Nan and Cao 2023) and a total of 3.25 billion m^3^ of water will be continuously diverted to the Yongding River in 2024.

The ongoing ecological water replenishment has significantly altered the riverscape and physicochemical parameters of water (Zhu et al. 2021), reshaping the water ecological environment of the Yongding River (Bai et al. 2023, Nan and Cao 2023). In this context, the population structure and diversity of fish will change accordingly and fish invasion may also occur more easily (Schmidt et al. 2019, Guo et al. 2020, Gao et al. 2023). Thus, data for the overall baseline fish biodiversity before large-scale water replenishment will enable researchers to better understand how water replenishment affects fish biodiversity and the ecological environment. In order to acquire the fish diversity data in the Yongding River Basin, two field collections in the summer and autumn of 2019 were conducted. The provided data attempt to help other researchers evaluate the effects of water replenishment on fish communities in the Yongding River Basin.

## Sampling methods

### Sampling description

Fish specimens were collected at 46 sites in the Yongding River Basin in 2019 (Fig. 1). We sampled various aquatic environments (mountainous streams, plain river channels and reservoirs) using a combination of hand nets (dense mesh), cast nets (aperture 1 cm × 1 cm, diameter 5 m, length 3.5 m) and traps (aperture 0.5 cm × 0.5 cm, length 10 m). Hand nets and cast nets were used in fast-flowing and shallow waters, while traps were employed in slow-flowing waters and pools. Four traps were usually placed at the sampling point overnight for 12 hours. When hand nets served as the primary collection tool, we sampled each site for one hour. When cast nets were used, each sampling point was sampled for 30 minutes.. The specimens were fixed in 95% ethanol or 10% formaldehyde solution in the field. The coordinates of sampling locations were recorded using a hand-held GPS locator. The specimens were stored in 95% ethanol at the National Zoological Museum, Institute of Zoology, Chinese Academy of Sciences (ASIZB).

### Step description

Fish species were identified and referred to relevant published literature of the Yongding River and its surrounding rivers (Zhang et al. 2011). Valid species names were in accordance with the taxonomic literature (Zhang et al. 2020).

## Geographic coverage

### Description

We surveyed the entire main stream and nearly all the tributaries of the Yongding River Basin (Fig. 1). Rivers covered various habitats, including swift-flowing waters, running waters and pools. The collection sites were marked using ArcGIS 10.2 software.

### Coordinates

38.8805 and 40.6604 Latitude; 112.1643 and 117.0914 Longitude.

## Taxonomic coverage

### Description

In total, nine orders, 18 families, seven subfamilies, thirty-three genera, and forty-five species were collected in the Yongding River and its tributaries.

### Taxa included

**Table:** 

Rank	Scientific Name	
kingdom	Animalia	
phylum	Chordata	
class	Osteichthyes	
order	Anabantiformes	
family	Channidae	
genus	*Channa* Scopoli, 1777	
species	*Channaargus* (Cantor, 1842)	
family	Osphronemidae	
subfamily	Macropodusinae	
genus	*Macropodus* Lacepède, 1801	
species	*Macropoduschinensis* (Bloch, 1790)	
order	Beloniformes	
family	Adrianichthyidae	
subfamily	Oryziinae	
genus	*Oryzias* Jordan & Snyder, 1906	
species	*Oryziaslatipes* (Temminck & Schlegel, 1846)	
order	Cypriniformes	
family	Acheilognathidae	
genus	*Acheilognathus* Bleeker, 1859	
species	*Acheilognathuschankaensis* (Valenciennes, 1844)	
species	*Acheilognathusmacropterus* (Bleeker, 1871)	
species	*Acheilognathusrhombeus* (Temminck & Schlegel, 1846)	
genus	*Rhodeus* Agassiz, 1832	
species	*Rhodeusnotatus* Nichols, 1929	
species	*Rhodeusocellatus* (Kner, 1866)	
species	*Rhodeussinensis* Günther, 1868	
family	Cobitidae	
genus	*Cobitis* Linnaeus, 1758	
species	*Cobitismelanoleuca* Nichols, 1925	
genus	*Misgurnus* Lacepède, 1803	
species	*Misgurnusanguillicaudatus* (Cantor, 1842)	
species	*Misgurnusdabryanus* Dabry de Thiersant, 1872	
family	Cyprinidae	
subfamily	Cyprininae	
genus	*Carassius* Jarocki, 1822	
species	*Carassiusauratus* (Linnaeus, 1758)	
genus	*Cyprinus* Linnaeus, 1758	
species	*Cyprinuscarpio* Linnaeus, 1758	
family	Gobionidae	
genus	*Abbottina* Jordan & Fowler, 1903	
species	*Abbottinarivularis* (Basilewsky, 1855)	
genus	*Gobio* Cuvier, 1816	
species	*Gobiorivuloides* Nichols, 1925	
genus	*Pseudorasbora* Bleeker, 1860	
species	*Pseudorasboraparva* (Temminck & Schlegel, 1846)	
genus	*Sarcocheilichthys* Bleeker, 1860	
species	*Sarcocheilichthyssciistius* (Abbott, 1901)	
family	Leuciscidae	
subfamily	Leuciscinae	
genus	*Leuciscus* Cuvier, 1816	
species	*Leuciscuswaleckii* (Dybowski, 1869)	
subfamily	Pseudaspininae	
genus	*Rhynchocypris* Günther, 1889	
species	*Rhynchocyprislagowskii* (Dybowski, 1869)	
species	*Rhynchocyprisoxycephalus* (Sauvage & Dabry de Thiersant, 1874)	
family	Nemacheilidae	
genus	*Barbatula* Linck, 1790	
species	*Barbatulanuda* (Bleeker, 1869)	
genus	*Lefua* Herzenstein, 1888	
species	*Lefuacostata* (Kessler, 1876)	
genus	*Triplophysa* Rendahl, 1933	
species	*Triplophysadalaica* (Kessler, 1876)	
family	Xenocyprididae	
genus	*Chanodichthys* Bleeker, 1860	
species	*Chanodichthyserythropterus* (Basilewsky, 1855)	
genus	*Ctenopharyngodon* Steindachner, 1866	
species	*Ctenopharyngodonidella* (Valenciennes, 1844)	
genus	*Hemiculter* Bleeker, 1860	
species	*Hemiculterbleekeri* Warpachowski, 1887	
species	*Hemiculterleucisculus* (Basilewsky, 1855)	
genus	*Hypophthalmichthys* Bleeker, 1860	
species	*Hypophthalmichthysmolitrix* (Valenciennes, 1844)	
species	*Hypophthalmichthysnobilis* (Richardson, 1845)	
genus	*Opsariichthys* Bleeker, 1863	
species	*Opsariichthysbidens* Günther, 1873	
genus	*Zacco* Jordan & Evermann, 1902	
species	*Zaccoplatypus* (Temminck & Schlegel, 1846)	
order	Gobiiformes	
family	Gobiidae	
subfamily	Gobionellinae	
genus	*Rhinogobius* Gill, 1859	
species	*Rhinogobiusbrunneus* (Temminck & Schlegel, 1845)	
species	*Rhinogobiuscliffordpopei* (Nichols, 1925)	
species	*Rhinogobiussimilis* Gill, 1859	
family	Odontobutidae	
genus	*Micropercops* Fowler & Bean, 1920	
species	*Micropercopsswinhonis* (Günther, 1873)	
genus	*Odontobutis* Bleeker, 1874	
species	*Odontobutispotamophila* (Günther, 1861)	
order	Osmeriformes	
family	Osmeridae	
genus	*Hypomesus* Gill, 1862	
species	*Hypomesusolidus* (Pallas, 1814)	
order	Perciformes	
family	Gasterosteidae	
genus	*Pungitius* Coste, 1846	
species	*Pungitiussinensis* (Guichenot, 1869)	
order	Salmoniformes	
family	Salmonidae	
subfamily	Salmoninae	
genus	*Oncorhynchus* Suckley, 1861	
species	*Oncorhynchusmykiss* (Walbaum, 1792)	
order	Siluriformes	
family	Bagridae	
genus	*Tachysurus* Lacepède, 1803	
species	*Tachysurussinensis* Lacepède, 1803	
species	*Tachysurusvachellii* (Richardson, 1846)	
family	Siluridae	
genus	*Silurus* Linnaeus, 1758	
species	*Silurusasotus* (Linnaeus, 1758)	
species	*Silurusmeridionalis* Chen, 1977	
order	Synbranchiformes	
family	Mastacembelidae	
genus	*Sinobdella* Kottelat & Lim, 1994	
species	*Sinobdellasinensis* (Bleeker, 1870)	

## Temporal coverage

**Data range:** 2019-6-04 – 2019-11-17.

## Usage licence

### Usage licence

Other

### IP rights notes

Creative Commons Attribution License (CC BY 4.0)

## Data resources

### Data package title

Collected fish taxon-occurrences of the Yongding River Basin, China.

### Resource link

https://doi.org/10.15468/3dhqn5

### Number of data sets

1

### Data set 1.

#### Data set name

Collected fish taxon-occurrences of the Yongding River Basin, China.

#### Data format

Darwin Core

#### Download URL

http://ipt.pensoft.net/archive.do?r=y-d

#### Description

The dataset presents 45 fish detected in the Yongding River Basin, with a total of 718 data records and the number of fish being 6,238 (Li and Chen 2024). Important information, including taxonomic, geographic location of the occurrence, water body and event date, was provided for 45 fish species.

**Data set 1.** 

Column label	Column description
occurrenceID	An identifier for the dwc:Occurrence (as opposed to a particular digital record of the dwc:Occurrence). In the absence of a persistent global unique identifier, construct one from a combination of identifiers in the record that will most closely make the dwc:occurrenceID globally unique.
catalogNumber	An identifier (preferably unique) for the record within the dataset or collection.
basisOfRecord	Recommended best practice is to use a controlled vocabulary such as the set of local names of the identifiers for classes in Darwin Core.
collectionCode	The name, acronym, coden or initialism identifying the collection or dataset from which the record was derived.
eventDate	The date-time or interval during which a dwc:Event occurred. For occurrences, this is the date-time when the dwc:Event was recorded. Not suitable for a time in a geological context.
samplingProtocol	The methods or protocols used during a dwc:Event, denoted by an IRI.
countryCode	The standard code for the country in which the dcterms:Location occurs.
scientificName	The full scientific name, with authorship and date information if known. When forming part of a dwc:Identification, this should be the name in the lowest level taxonomic rank that can be determined. This term should not contain identification qualifications, which should instead be supplied in the dwc:identificationQualifier term.
kingdom	The full scientific name of the kingdom in which the dwc:Taxon is classified.
phylum	The full scientific name of the phylum or division in which the dwc:Taxon is classified.
class	The full scientific name of the class in which the dwc:Taxon is classified.
order	The full scientific name of the order in which the dwc:Taxon is classified.
family	The full scientific name of the family in which the dwc:Taxon is classified.
subfamily	The full scientific name of the subfamily in which the dwc:Taxon is classified.
genus	The full scientific name of the genus in which the dwc:Taxon is classified.
taxonRank	The taxonomic rank of the most specific name in the dwc:scientificName.
ownerInstitutionCode	The name (or acronym) in use by the institution having ownership of the object(s) or information referred to in the record.
individualCount	The number of individuals present at the time of the dwc:Occurrence.
recordedBy	A person, group or organisation responsible for recording the original dwc:Occurrence.
identifiedBy	A list (concatenated and separated) of names of people, groups or organisations who assigned the dwc:Taxon to the subject.
decimalLatitude	The geographic latitude (in decimal degrees, using the spatial reference system given in dwc:geodeticDatum) of the geographic centre of a dcterms:Location. Positive values are north of the Equator, negative values are south of it. Legal values lie between -90 and 90, inclusive.
decimalLongitude	The geographic longitude (in decimal degrees, using the spatial reference system given in dwc:geodeticDatum) of the geographic centre of a dcterms:Location. Positive values are east of the Greenwich Meridian, negative values are west of it. Legal values lie between -180 and 180, inclusive.
maximumElevationInMetres	The upper limit of the range of elevation (altitude, usually above sea level), in metres.
minimumElevationInMetres	The lower limit of the range of elevation (altitude, usually above sea level), in metres.
geodeticDatum	The ellipsoid, geodetic datum or spatial reference system (SRS) upon which the geographic coordinates given in dwc:decimalLatitude and dwc:decimalLongitude are based.
coordinateUncertaintyInMetres	The horizontal distance (in metre) from the given dwc:decimalLatitude and dwc:decimalLongitude describing the smallest circle containing the whole of the dcterms:Location. Leave the value empty if the uncertainty is unknown, cannot be estimated or is not applicable (because there are no coordinates). Zero is not a valid value for this term.
coordinatePrecision	A decimal representation of the precision of the coordinates given in the dwc:decimalLatitude and dwc:decimalLongitude.
county	The full, unabbreviated name of the next smaller administrative region than stateProvince (county, shire, department etc.) in which the dcterms:Location occurs.
country	The name of the country or major administrative unit in which the dcterms:Location occurs.
stateProvince	The name of the next smaller administrative region than country (state, province, canton, department, region etc.) in which the dcterms:Location occurs.
municipality	The full, unabbreviated name of the next smaller administrative region than county (city, municipality etc.) in which the dcterms:Location occurs. Do not use this term for a nearby named place that does not contain the actual dcterms:Location.
waterBody	The name of the waterbody in which the dcterms:Location occurs.
establishmentMeans	Statement about whether a dwc:Organism has been introduced to a given place and time through the direct or indirect activity of modern humans.
degreeOfEstablishment	The degree to which a dwc:Organism survives, reproduces and expands its range at the given place and time.
samplingEffort	The amount of effort expended during a dwc:Event.

## Additional information

**Figure 2.**
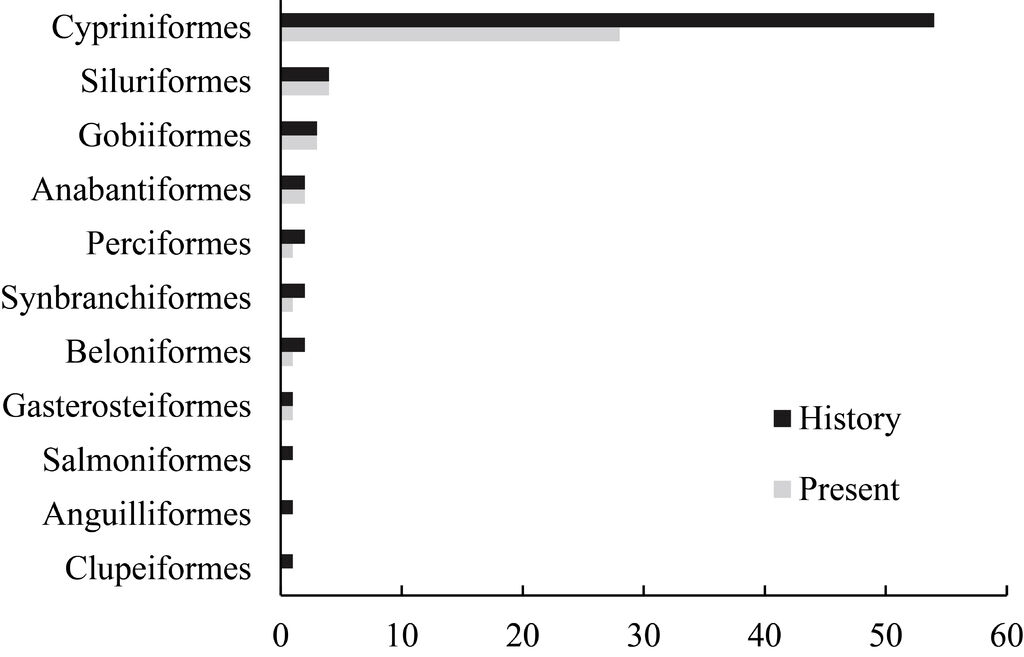
Historical changes of native fish species in the Yongding River.

A total of 45 fish species were collected during our field surveys, of which 41 are native fish species. These 41 native species belong to seven orders, 16 families, six subfamilies and 30 genera. The native fish composition of the Yongding River is dominated by Cypriniformes (29 species), followed by Gobiiformes (five species) and Siluriformes (four species). Native species are relatively evenly distributed at the family level, with Acheilognathidae being the most diverse family (six species), followed by Xenocypridae and Gobionidae, each with four species. Additionally, four non-native fish species were collected, belonging to three orders, four families and four genera. These include *Misgurnusdabryanus*, *Odontobutispotamophilus*, *Hypomesusolidus* and *Oncorhynchusmykiss*. According to historical research (Fig. 2), which includes fish species data from the Yongding River up to 2019, there were 73 native fish species, of which 32 species were not found in our study (Zhang et al. 2011).

The Middle Yellow River serves as the main source of ecological water replenishment for the Yongding River, where ongoing water replenishment efforts are leading to changes in the aquatic environment (Li et al. 2022). River sections that had been dry for many years are being restored through simple water replenishment techniques. Out of the 73 fish species in the Yongding River, 58 species are shared with the Middle Yellow River (Zhao et al. 2020), representing approximately 79.45% of the total species in the Yongding River. It suggests that diverting water from the Middle Yellow River to the Yongding River may be ecologically suitable. However, it remains uncertain whether native fish diversity and populations will fully recover or if the number of non-native fish species will continue to rise. Further field studies are needed to confirm these potential changes.
